# Both ischemic preconditioning and ghrelin administration protect hippocampus from ischemia/reperfusion and upregulate uncoupling protein-2

**DOI:** 10.1186/1472-6793-9-17

**Published:** 2009-09-22

**Authors:** Yajun Liu, Lianbi Chen, Xiaoqun Xu, Eric Vicaut, Richard Sercombe

**Affiliations:** 1 Institute of Physiology, School of Medicine, Shandong University, Jinan 250012, Shandong, PR China; 2 Laboratory of Microcirculation Research (EA 3509), University Paris 7, France; 3 Institute of Basic Medicine, Shandong Academy of Medical Sciences, Jinan 250012, Shandong, PR China; 4 Medical Pharmacology and Physiology, School of Medicine, University of Missouri, 1 Hospital drive, Columbia, MO 65212, USA

## Abstract

**Background:**

A major endogenous protective mechanism in many organs against ischemia/reperfusion (I/R) injury is ischemic preconditioning (IPC). By moderately uncoupling the mitochondrial respiratory chain and decreasing production of reactive oxygen species (ROS), IPC reduces apoptosis induced by I/R by reducing cytochrome c release from the mitochondria. One element believed to contribute to reduce ROS production is the uncoupling protein UCP2 (and UCP3 in the heart). Although its implication in IPC in the brain has been shown in vitro, no in vivo study of protein has shown its upregulation. Our first goal was to determine in rat hippocampus whether UCP2 protein upregulation was associated with IPC-induced protection and increased ROS production. The second goal was to determine whether the peptide ghrelin, which possesses anti-oxidant and protective properties, alters UCP2 mRNA levels in the same way as IPC during protection.

**Results:**

After global forebrain ischemia (15 min) with 72 h reperfusion (I/R group), we found important neuronal lesion in the rat hippocampal CA1 region, which was reduced by a preceding 3-min preconditioning ischemia (IPC+I/R group), whereas the preconditioning stimulus alone (IPC group) had no effect. Compared to control, UCP2 protein labelling increased moderately in the I/R (+39%, NS) and IPC+I/R (+28%, NS) groups, and substantially in the IPC group (+339%, P < 0.05). Treatment with superoxide dismutase (10000 U/kg ip) at the time of a preconditioning ischemia greatly attenuated (-73%, P < 0.001) the increase in UCP2 staining at 72 h, implying a role of oxygen radicals in UCP2 induction.

Hippocampal UCP2 mRNA showed a moderate increase in I/R (+33%, P < 0.05) and IPC+I/R (+40%, P < 0.05) groups versus control, and a large increase in the IPC group (+333%, P < 0.001). In ghrelin experiments, the I/R+ghrelin group (3 daily administrations) showed considerable protection of CA1 neurons versus I/R animals, and increased hippocampal UCP2 mRNA (+151%, P < 0.001).

**Conclusion:**

We confirm that IPC causes increased expression of UCP2 protein in vivo, at a moment appropriate for protection against I/R in the hippocampus. The two dissimilar protective strategies, IPC and ghrelin administration, were both associated with upregulated UCP2, suggesting that UCP2 may often represent a final common pathway in protection from I/R.

## Background

Protection against ischemic lesion has been very extensively studied in the heart and brain. One powerful endogenous mechanism of protection present in these and other organs is ischemic preconditioning. This consists of a single or a series of brief, non-lethal ischemic periods which condition the tissue to resist against significant cell death when subsequently challenged by a normally lethal ischemia. Delayed protection, which is the subject of the present study, takes place from 12 h to 7 days after the preconditioning ischemia [1-5], and must involve transcriptional regulation.

Ischemia of cardiac cells or neurons leads to apoptosis which occurs via release of cytochrome c from mitochondria [6-10]. This activates the caspase cascade. These events are triggered in large part by excess mitochondrial reactive oxygen species (ROS) [7,11-13].

In the heart, as in the brain, ischemic tolerance induced by preconditioning is associated with modest uncoupling of the mitochondrial respiratory chain [2,3], which reduces the production of ROS by respiration. A variety of signalling pathways seems to be involved. One of the mitochondrial elements believed to play a role in such preconditioning are the uncoupling proteins UCP2 and UCP3. There is clear evidence of increased expression of UCP2/3 in the heart in this situation [2,14], and more limited evidence in the brain for UCP2 [15,16] (only UCP2 is clearly expressed in the brain). In the brain, it has been shown that new protein expression can be triggered by large-scale mitochondrial ROS production occurring at reperfusion [17,18].

With respect to the brain, the uncoupling protein, UCP-2, has been recently noted to possess a certain neuroprotective activity [15,16,19,20]. Situated in the inner mitochondrial membrane, it is distributed in several brain regions [19,21]. Its role appears to be to dissipate the proton electrochemical gradient through the mitochondrial inner membrane [22,23]. By this means, it mildly uncouples oxidative phosphorylation from respiration, decreases the inner membrane potential, and reduces ROS production, especially superoxide, by the respiratory complexes [24,25]. Thus, increased expression of this protein coincident with ischemia should reduce the production of ROS in mitochondria and confer protection on cells subject to I/R. Compatible effects of UCP2 have indeed been demonstrated in in vitro preparations [15,16], but only the study by Mattiasson et al [15] has so far provided in vivo data, in the form of measures of UCP2 mRNA, compatible with this possibility in ischemic preconditioning. Increased protein expression has not yet been demonstrated.

The peptide ghrelin is an endogenous ligand for the growth hormone secretagogue (GHS) receptor (GHS-R) [26]. Although its role in the control of feeding and energy metabolism is well known, it also exerts protective effects against I/R injury in the cardiovascular system [27-29] and the gastro-intestinal system [30-32]. An inhibitory influence on apoptosis has been reported [28,33].

We recently reported for the first time that post-ischemic ghrelin administration was protective and anti-apoptotic in the brain, in rat hippocampus, after global forebrain I/R [34]. Subsequently, it has been shown that ghrelin-induced neuroprotection in vitro (glucose-deprivation lesion) was associated with inhibition of the mitochondrial apoptosis pathway (cytochrome c release and caspase-3 activation), and increased Bcl2/Bax ratio [35]. These phenomena are associated with reduced I/R injury to the hippocampal CA1 neurons after IPC [6,8,36]. Another recent study has confirmed that redox injury and apoptotic mechanisms could be inhibited by ghrelin in cortical neurons subject to I/R [37].

Thus, although apparently different in their modes of triggering protection, IPC and ghrelin exert similar strong protection in the brain, including the hippocampus. Both are also capable of protecting other organs such as heart and intestine.

Concerning ghrelin, Chung et al [35] also showed that this protection was associated with mitochondrial membrane potential stabilization and decreased ROS formation, compatible with increased activity of UCP2. This suggests, therefore, that neuroprotection by either ghrelin or IPC could involve inhibition of the mitochondrial apoptosis pathway through increased expression of UCP2. Indeed, it has been shown in different tissues that ghrelin can induce upregulation of UCP2 and sometimes UCP3 [38,39]. Furthermore, a recent study in the heart showed that although IPC can involve different signalling pathways, these converge finally at the mitochondrial respiratory pathway [13].

The experiments undertaken in this study sought to confirm that UCP2 protein was effectively over-expressed in vivo in ischemically preconditioned hippocampus, and to determine if the production of ROS could be responsible for this increased expression. Second, by similar measurements of UCP2 mRNA, we determined whether IPC and ghrelin administration involved similar changes in UCP2 protein transcription when used to attenuate hippocampal ischemic lesion. Our results confirm the increased UCP2 expression and reveal a striking resemblance in the final action of ghrelin and IPC.

## Results

### UCP2 protein expression in hippocampal neurons after IPC

**Figure 1:**
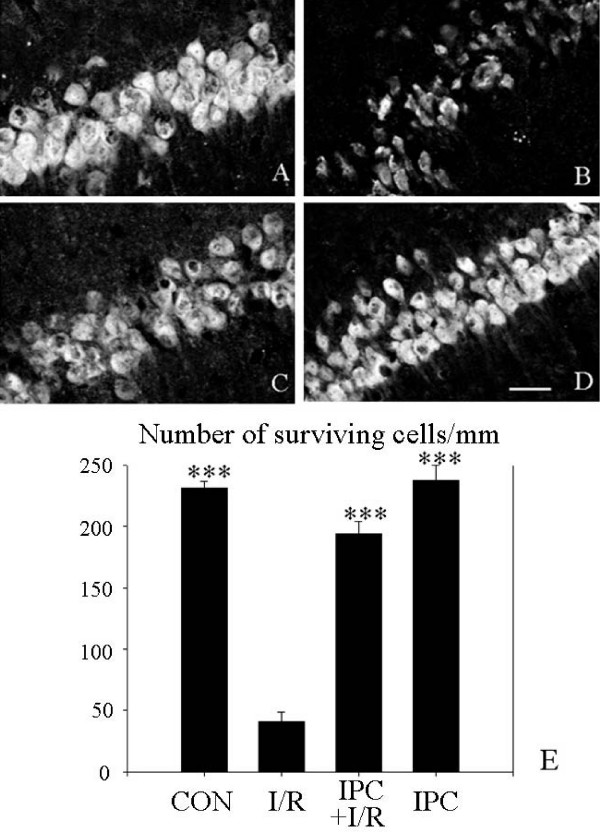
**Ischemic preconditioning reduces the neuronal damage of the hippocampal CA1 region in rats (confocal microscope images of anti-NeuN staining by indirect immunohistochemistry)**. A: control group; B: I/R group; C: IPC+ I/R group; D: IPC group. Scale bar = 30 μm; E: bar graph of results of neuronal counting (performed blinded) expressed as number of intact neurons/mm CA1 pyramidal layer (mean ± S.E.M, n = 4 animals). Significant differences were determined by repeated measures ANOVA plus Tukey's test. *** P < 0.001 compared to I/R group.

**Figure 2:**
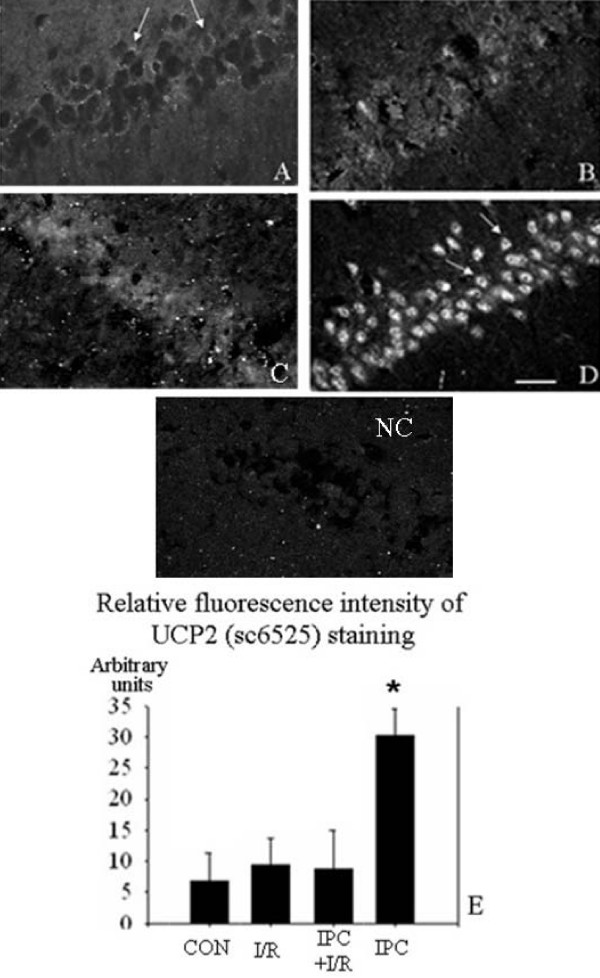
**Expression of UCP-2 in CA1 area of hippocampus (confocal microscope images of UCP2 staining by indirect immunohistochemistry) as observed with sc6525 (A, B, C, D) anti-UCP2 (C-terminal antibody)**. A, control; B, I/R; C, IPC+I/R; D, IPC. NC, negative control obtained in the absence of the anti-UCP2. Scale bar = 30 μm. E: Bar graph of results of UCP-2 immunofluorescence (sc6525 images) in the CA1 pyramidal layer, expressed as average intensity after subtraction of background fluorescence (mean ± S.E.M, n = 4 animals). Significant differences were determined by repeated measures ANOVA plus Tukey's test. * P < 0.05 compared to all other groups.

**Figure 3:**
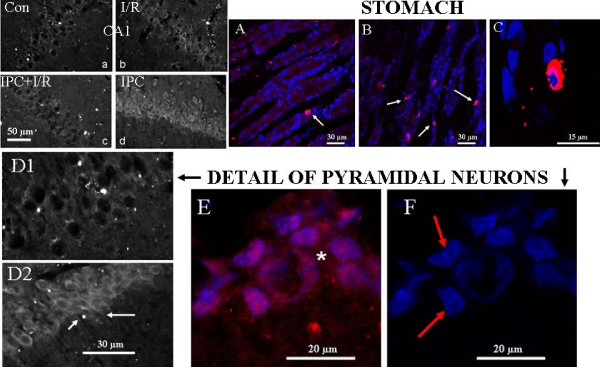
**Control experiments**. a, b, c, d, expression of UCP-2 in CA1 area of hippocampus (confocal microscope images of UCP2 staining by indirect immunohistochemistry) in examples of the 4 groups indicated, as observed with sc6526 (N-terminal antibody). A, B, C, indirect immunohistochemical labeling of UCP2 (red) in stomach (A, B, C). The DNA is stained blue with DAPI. White arrows in A and B indicate some of the UCP2-positive cells of the mucosal layer. Typically, there are more in the stomach from a fasted animal (B) than from that of a control animal (A). C shows detail of one cell, with an overlapping zone (pink) of UCP2 immunoreactivity and DAPI. D1, D2, CA1 neurons of the hippocampus showing pyramidal cells from a control animal and an IPC animal respectively. Notice the proximal dendritic staining of UCP2 in the highly stained IPC animal (arrows in D2). E, F, high power fields of pyramidal cells in an IPC animal, E with UCP2 staining and F without UCP2 staining. Notice the difference in the apparent shape and size of pyramidal cell nuclei between control (D1, round unstained nuclei) and IPC animals (F, polygonal DAPI-stained nuclei, red arrows). Widespread homogeneous UCP2 immunoreactivity was present in the pyramidal cell cytoplasm in the IPC group (E, star); it also appeared interspersed, non-homogeneously, with the DAPI staining. UCP2 staining with sc6525 except in D (sc6526).

1. Ischemic preconditioning protects CA1 neurons. The protection afforded by IPC to the CA1 area of the hippocampus was assessed with anti-NeuN staining. NeuN is a specific, neuronal protein. Typical images from brains of the four groups are shown in Fig 1. The I/R group (Fig 1B) showed a majority of neurons with much reduced, irregular staining, compared to the three other groups, which appeared very similar. Thus, the preconditioning stimulus did not itself cause a significant reduction in normal neurons. Blinded counting of intact neurons gave the result shown in Fig 1E, indicating a high degree of neuronal protection in the IPC+I/R group.

2. UCP2 protein immunoreactivity is increased in CA1 neurons after ischemic preconditioning. The variation of UCP2 protein expression in CA1 pyramidal neurons was examined by IHC. Two anti-UCP2 antibodies were tested in the hippocampi of the four groups. Typical results are shown in Fig 2, obtained with a C-terminal antibody (sc6525). The general pattern of results found in each series was very similar, showing very weak staining in the controls (A), slightly brighter staining in the I/R (B) and I/R+IPC (C) groups, and much stronger staining in the IPC group (D). Since the sc6525 antibody gave stronger staining, we used it for the statistical comparison of the fluorescence intensity in the four groups. Absence of staining was found when this antibody was omitted (Fig 2NC). The graph (Fig 2E) shows that the intensity in the IPC group considerably exceeded that of the other three groups. The fluorescence intensities of the I/R and IPC+I/R groups were numerically higher than, but not significantly different from, those from control. This evaluation comprised a correction for the differences in background fluorescence (see methods).

3. Further control experiments. Since doubts about the specificity and sensitivity of certain anti-UCP2 antibodies have been expressed [40,41], we performed other control experiments and examined the staining in detail. The N-terminal antibody sc 6526 was tested under identical conditions to sc6525 and found to stain the four types of hippocampal sections with an exactly similar pattern (Fig 3a, b, c, d). In stomach tissue from two control rats and three rats which fasted for 24 h, we found UCP2 immunoreactivity in 5-6 times more cells in the fasting animals (Fig 3B) than in controls (Fig 3A). Fasting has previously been shown to increase the UCP2 protein detected by western blot in the stomach of mice [40]. Fig 3C is an enlargement of one cell positive for UCP2 immunoreactivity. It reveals a close relation of UCP2 immunoreactivity and DAPI staining (pink zone).

Whereas UCP2 staining in the control situation was weak and limited to a perinuclear halo (Fig 3D1), in some cases in the IPC group it was observed to invade the proximal part of the pyramidal cell dendrites (Fig 3D2), as previously noted [16]. In the IPC-treated brains, in which the the UCP2 immunoreactivity was always far denser, the staining pervaded a large part of the cytoplasm and often appeared intimately related to the DAPI staining of DNA in the nucleus (Fig 3E, F), the shape of which became less regular (cf panel D1). Outside the pyramidal cell layer, small DAPI-stained nuclei of presumed glial cells were not associated with UCP2 staining (not shown). In the cytoplasm of pyramidal neurons, a modest degree of overlap with NeuN immunoreactivity was noted (not shown).

**Figure 4:**
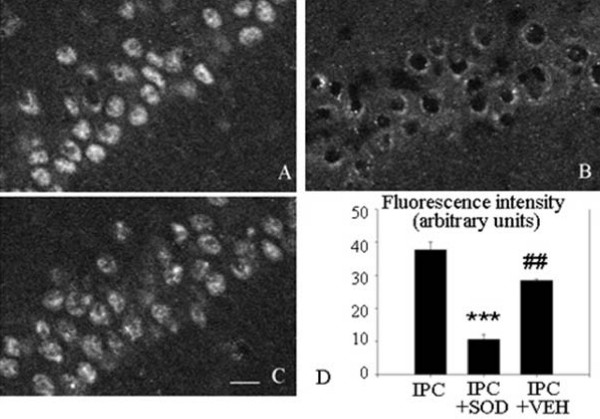
**Effects of SOD treatment on UCP2 immunoreactivity (indirect method) in the hippocampal CA1 area**. Confocal microscope images of UCP2 immunoreactivity of A: IPC group; B: IPC + SOD group; C: IPC +vehicle group. Notice the similarity in the appearance of SOD-treated neurons in B with that of control neurons in Fig 2A. D: Bar graph of fluorescence intensities of UCP-2 staining (mean ± S.E.M, n = 4 animals). Scale bar A, B, C = 15 μm. Significant differences were determined by repeated measures ANOVA plus Tukey's test. *** P < 0.001 compared to IPC group; ## P < 0.01 compared to IPC + SOD group.

**Figure 5:**
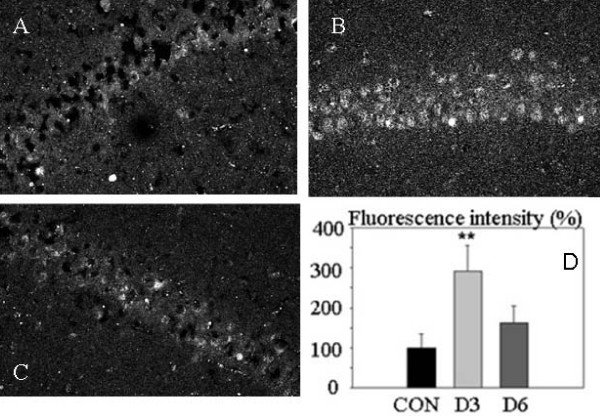
**Effects of post-conditioning ischemia time on UCP2 immunoreactivity in the hippocampal CA1 area**. Confocal microscope images of UCP2 immunoreactivity of A: control group; B: D3 group (3 days post-ischemia); C: D6 group (6 days post-ischemia). D: Bar graph of fluorescence intensities of UCP2 staining (mean ± SEM, n = 4 animals). Significant differences were determined by repeated measures ANOVA plus Tukey's test. ** P < 0.05 compared to Control or D6. Scale bar E, F, G = 50 μm.

4. Increase in UCP2 immunoreactivity in IPC brains is blocked by ROS scavenging. In three groups of rats undergoing IPC treatment, one group received no treatment, one received intraperitoneal SOD and one received only vehicle. As shown in Fig 4A, B, C, D, the brains from the SOD-treated rats showed a dramatic reduction in the UCP2 immunoreactivity detected at day 3. Indeed, even the morphological aspect of the remaining UCP2 immunoreactivity (Fig 4B) resembled that seen in the non-ischemic animals (Fig 2A or 3a).

5. The level of UCP2 immunoreactivity fades considerably between 3 and 6 days after IPC. When groups of rats subjected to IPC were sacrificed at day 6, the UCP2 immunoreactivity had decreased significantly to a low level compared to the high level observed at day 3 (Fig 5B, C). Thus, a high level of expression of UCP2 was seen at day 3, whereas, although numerically higher, the fluorescence intensity at day 6 was not significantly different from the control (Fig 5D).

### Comparison of UCP2 mRNA in IPC- and ghrelin-treated hippocampi

**Figure 6:**
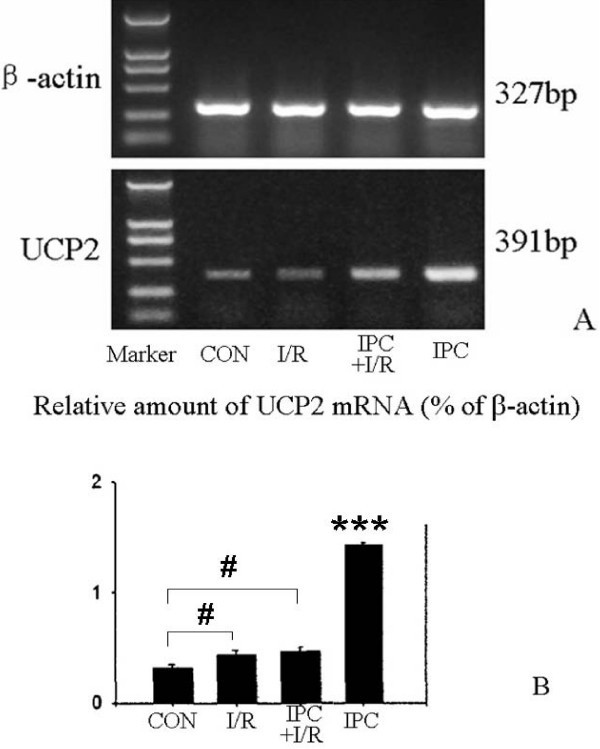
**Effects of various treatments on UCP-2 mRNA expression in IPC experiments**. A: RT-PCR of UCP-2 mRNA in the hippocampal preparations in Control, I/R, IPC+I/R and IPC groups. B: Bar graph of mean results (± SEM) of RT-PCR determinations of UCP-2 mRNA in hippocampal preparations, expressed relative to the quantity of β-actin mRNA (=100%). n = 6 animals. Significant differences were determined by repeated measures ANOVA plus Tukey's test. *** P < 0.001 compared to all other groups; # P < 0.05 compared to CON group.

**Figure 7:**
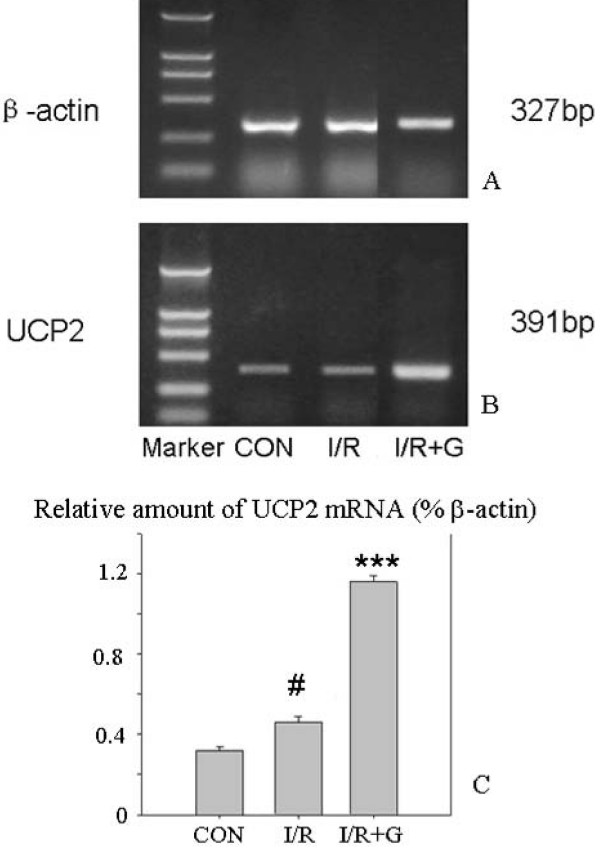
**Effects of various treatments on UCP-2 mRNA expression in ghrelin experiments**. RT-PCR of UCP2 mRNA in the hippocampal preparations in the control group, I/R group (vehicle alone), or I/R group treated with ghrelin (G). A, B: Typical bands obtained for, respectively, β-actin and UCP-2 mRNA. C: Quantitative results expressed with respect to β-actin mRNA in each of the three groups. n = 6 animals. *** significantly different from the control and I/R groups, P < 0.001. # significantly different from the control group, P < 0.05. ANOVA + Tukey's test.

1. Ischemic preconditioning increases the amount of hippocampal UCP2 mRNA. In a set of parallel experiments, hippocampi from the same four groups as in the IHC experiments were compared by RT-PCR to compare the amounts of UCP2 mRNA present (Fig 6). Fig 6A illustrates a typical gel, the overall results being shown in Fig 6B. mRNA was increased considerably in the IPC group, but only moderately (though significantly) in the I/R and IPC+I/R groups, compared to control. The two intermediate groups were also significantly different from the IPC group. This result suggests that a high level of transcription of mRNA was induced in the hippocampus by the IPC treatment. However, in the IPC+I/R group, at the time of the sacrifice 6 days after IPC, the level had fallen back to the level of the I/R group.

2. Ghrelin treatment is accompanied by increased amounts of hippocampal UCP2 mRNA. Fig 7 shows that the I/R group treated with ghrelin on days 0, 1, and 2 contained significantly increased amounts of UCP2 mRNA in the hippocampus on day 3, indicating that increased UCP2 transcription was associated with the protection of hippocampal neurons. This result is to be set against our previous results on ghrelin-induced protection: intraperitoneal ghrelin on days 0, 1 and 2 afforded a strong protection of the CA1 neurons against our standard ischemia, as measured by cell counting in hematoxylin and eosin-stained sections [34].

Table 1 compares the overall results on CA1 neuroprotection and hippocampal UCP2 mRNA for IPC and ghrelin treatment. Both treatments were associated with an augmented level of UCP2 mRNA (columns 3 and 7), although this was relatively lower in the IPC+I/R group (column 4), in which the animals were killed on day 6.

**Table 1:** Comparison of numbers of surviving CA1 neurons and relative amounts of hippocampal UCP-2 mRNA determined in the various groups.

	**----------IPC experiment ----------**				**---Ghrelin experiment---**		
**GROUP**	**1)CON**	**2)I/R**	**3)IPC**	**4)IPC+I/R**	**5)CON**	**6)I/R**	**7)I/R+G**

Number of surviving neurons/mm (n = 4/6)	231.6 ± 5.2	41.1^a ^± 7.7	237.8 ± 12.1	194.1 ± 9.8	**251.3 ± 8.7**	**28.5^a ^± 7.3**	**179.5^b, c ^± 11.5**

Relative amount (%) of UCP-2 mRNA (n = 6/4)	0.31 ± 0.03	0.44^b ^± 0.04	1.45^a ^± 0.02	0.46^b ^± 0.04	0.32 ± 0.02	0.46^b ^± 0.03	1.16^a ^± 0.04

Day of sacrifice	D3	D3	D3	D6	D3	D3	D3

mRNA expressed relative to that of β-actin. Day of sacrifice with respect to the first ischemia or sham ischemia (D0). Left 4 columns are set of measurements from IPC experiments; right 3 columns are set of measurements from ghrelin experiments. **Bold characters**: Values taken from Liu et al [34]. **a**: significantly different from all other groups, P < 0.001. **b**: significantly different from CON, P < 0.05. **c**: significantly different from I/R, P < 0.001. ANOVA + Tukey's test.

## Discussion

Only one previous publication has shown evidence of increased expression of brain UCP2, measured as mRNA by in situ hybridization, after preconditioning ischemia in vivo [15]. We have shown in vivo changes in UCP2 mRNA in parallel with changes in UCP2 protein expression in the I/R, IPC+I/R and IPC groups compared with control. Compatible in vitro effects (cell cultures) on UCP2 expression have been found by Diano et al [16], and Mattiasson et al [15]. Our results concerning a global forebrain ischemia model provide more complete in vivo data on UCP2 protein expression by immunohistochemistry, confirm and extend the mRNA measurements, extend the data on temporal aspects of UCP2 expression, and demonstrate its dependence on ROS production.

Furthermore, our experiments on mRNA establish for the first time a similar relationship between two dissimilar strategies, IPC or ghrelin treatment, which protect against I/R lesion, and upregulation of a mitochondrial ion transporter protein, UCP2.

### IPC-induced neuroprotection and UCP2

Previous studies have clearly shown the neuronal death induced in the hippocampal CA1 area by global forebrain ischemia, and the protection afforded by IPC [6,8,42]. Similar findings in the heart concern cardiocytes [2]. We confirm these results and further show that the preconditioning ischemia does not alone cause significant neuronal damage (Fig 1D, E). In parallel, UCP2 mRNA (Fig 6) and protein expression (Fig 2) both show strong upregulation of UCP2 after the same preconditioning ischemia (IPC group versus control). Several points need discussion.

*First*, one point of the overall paradigm that had to be optimized by compromise was the question of how long the reperfusion times should be. Barone et al [1] showed the preconditioning phenomenon to last 1-7 days and to require new protein formation before the lethal ischemia. Puisieux et al [43] confirmed a window of protection of at least 24-72 h with new protein formation. Schmidt-Kastner et al [44] studied the CA1 damage after a single long ischemia, and determined maximal CA1 damage at 48-72 h. Thus, we chose the delay of 3 days after IPC (until lethal ischemia or sacrifice) or after lethal ischemia (until sacrifice) because (a) it is sufficient to allow important formation of new protein between the preconditioning and the lethal ischemia, (b) it is within the range of efficacy of preconditioning, and (c) it is sufficient to allow evaluation of neuronal lesion after I/R.

*Second*, because of previous reports on UCP2 antibody problems (e.g. [40], we compared the antibody used for relative quantification (C-terminal peptide) with one raised against an N-terminal peptide (Fig 2 and 3). The general pattern of expression in the four groups was practically identical. It is difficult to conceive how two different molecules with appropriate peptide sequences (one C-terminal, one N-terminal) could behave in an apparently identical manner as observed by the two antibodies. However, this does not completely exclude the possibility that the one we used for quantification could bind to additional proteins. We tested it in the stomach and the clear-cut labelling of specific mucosal cells was multiplied several fold in fasting compared to control animals, exactly corresponding to the results of the western blot analysis of Pecqueur et al [40]. This increased expression of UCP2 could be linked to the upregulation of ghrelin in the stomach in fasting animals. We also checked the experiment with negative controls, i.e. absence of primary antibody (Fig 2NC). Other workers have considered these two antibodies to be specific to UCP2 [11,15,40,41,45,46], and Krauss et al [47] showed sc6525 to be unable to stain UCP2 knock-out mice.

*Third*, although the two measures of UCP2 upregulation gave parallel results (compare Figs 2E and 6B), the relative intergroup differences in values of mRNA/protein expression seem paradoxical, since the IPC+I/R group values are only modestly increased. The hypothesis we envisaged is that UCP2, which is known to reduce mitochondrial ROS production [23], would protect the neurons from a lethal ischemia if over-expressed during the appropriate period. This should be during the lethal ischemia and the subsequent reperfusion period. Our evidence is indeed that UCP2 upregulation was considerable at day 3 after a preconditioning ischemia, at the moment of induction of the lethal ischemia, as shown by both immunohistochemistry of UCP2 protein in the CA1 neurons and RT-PCR of UCP2 in the hippocampus. In contrast, at day 6 after a preconditioning ischemia alone (no following lethal ischemia) (Fig 5D), the UCP2 immunoreactivity had returned near to baseline. Thus, the time-course of expression after preconditioning is appropriately high at day 3 to protect the neurons during the phase of high ROS production, but at day 6 UCP2 over-expression is probably much reduced, which is compatible with the known duration of ischemic preconditioning [1,3]. In the case of preconditioned animals undergoing a second, lethal ischemia (IPC+I/R group), this lethal (15-min) ischemia presumably could not induce a large, durable surge of UCP2 synthesis, probably because the longer duration of ischemia induces a different pattern of response, which reduces protein synthesis [48]. Such was the case in the experiments of Chen et al [11], in which a 10-min lethal ischemia moderately and increasingly induced UCP2 protein expression (measured by western blot) from 2 to 18 h, after which it began to decline (24 h). In the experiments of Mattiasson et al [15], a significantly increased level of UCP2 mRNA was found in vivo at 48 h after a 3-min ischemia. A more detailed study of the time-course of ischemia-induced UCP2 upregulation is required to formulate a more precise hypothesis.

*Fourth*, there is important evidence that excess ROS production can induce UCP2 over-expression and activation [2,14,23,49,50]. Such a large-scale production of ROS occurs during ischemia, especially at reperfusion. When Mori et al [51] injected SOD at the time of a preconditioning ischemia (focal ischemia model), they observed strong attenuation of the protection against the subsequent lethal ischemia. We thus tested the hypothesis that UCP2 expression would not be upregulated if ROS were scavenged at the appropriate time, i.e. beginning at the time of the preconditioning ischemia. This experiment on the expression of UCP2 protein in IPC animals treated with SOD clearly confirmed that most of this increased expression is abolished. Moreover, this abolition of the increase in UCP2 immunoreactivity was associated with the reversion of the immunohistochemical image to the control pattern (Fig 4A-C), in which UCP2 labelling was limited to a weak perinuclear halo (Fig 2A).

Although SOD is a large molecule, its passage across the blood-brain barrier is facilitated by the ischemia itself which has been shown to alter the permeability of the microvessels [52]. Furthermore, systemic injection of SOD was used previously to prevent ROS production following global forebrain ischemia [53], a protocol which efficiently reduced ischemic damage. Our observations are strong evidence of the association of UCP2 over-expression with excess ROS production by the preconditioning ischemia. In contrast, it has been reported that a preconditioning ischemia does not induce upregulation of SOD, whether MnSOD or Cu-ZnSOD [44]. Overall, the present data are extremely coherent with an involvement of upregulated UCP2 in the IPC-induced protection of the CA1 area (and probably other hippocampal regions).

### Comparison of ghrelin- and IPC-induced effects on UCP2 mRNA in hippocampus

mRNA coding for receptors to ghrelin (GHS-1a receptors) have been found in brain, especially the hypothalamus, the hippocampus, and the anterior pituitary [26]. Both forms of ghrelin, acylated (octanoyl esterification at the serine residue 3) and unacylated, are known to cross the blood-brain barrier [33]. It seems established that only acylated ghrelin can bind to GHS receptors and release GH [26], but there may also be a direct, non-growth hormone (GH) releasing type of activity [27,28,33,54], presumably by another type of receptor. We administered the acylated form, but it is highly probable that plasmatic enzymes de-esterify the ser-octanoyl form and vice versa [55], so that we cannot distinguish which form is active here.

Neuroprotection by ghrelin was demonstrated by Liu et al [34] in this 4-vessel occlusion model (see Table 1), and by Chung et al [35] in a focal ischemia model. Both groups found inhibition of apoptosis in the hippocampus, and the latter group reported evidence of inhibition of the mitochondrial pathway (inhibition of mitochondrial cytochrome c release and caspase-3 activation, and increased Bcl2/Bax ratio). Ghrelin has also been shown to inhibit apoptosis in other models [28,33]. These anti-apoptosis mechanisms have also been demonstrated in IPC-induced neuroprotection [6,8,56]. It is probable therefore that anti-apoptosis mechanisms comprise a significant common pathway for neuroprotection by these two strategies. Furthermore, ghrelin has been shown to reduce oxidative stress in a rat seizure model [57], to reduce oxidative stress in the stomach after ischemia and to inhibit ROS generation in human polymorphonuclear leukocytes [31], and to inhibit ROS generation in hyperglycemic endothelial cells [58]. In the brain, it has recently been shown that the action of ghrelin on arcuate nucleus neurons is driven by a fatty acid oxidation pathway involving AMPK, CPT1 and free radicals that are scavenged by UCP2 [59]. Moreover, in other models, ghrelin administration has been shown to induce upregulation of UCP2 and UCP3, in white adipose tissue [38] and liver [39], and GH increases UCP2 mRNA in adipose tissue and skeletal muscle [60]. Such considerations led us to determine whether ghrelin administration and IPC induce similar upregulation of UCP2 mRNA in the hippocampus in a global I/R model.

In the ghrelin experiments, moderate upregulation of hippocampal UCP2 mRNA was seen (Fig 7) in the I/R group, similar to that found in the I/R group of the IPC series in Fig 6, and a much larger upregulation in the I/R+ghrelin group. We followed our previous protocol of 3 post-ischemic administrations at 24 h intervals which induced strong neuroprotection [34]. It is likely that this protocol led to a more persistent upregulation of UCP2 than would have been induced by a single injection of ghrelin or a single preconditioning ischemia.

Table 1 compares the neuroprotective and UCP2-inducing activities of IPC and ghrelin administration. Column 7 indicates that, in a lethal ischemia group, ghrelin increased both the number of surviving neurons and the measured amount of UCP2 mRNA. IPC alone (column 3) increased the measured amount of UCP2 mRNA (without significant reduction of the number of surviving neurons), but when it was followed by a lethal ischemia (column 4) the amount of UCP2 mRNA (on day 6) was reduced, though still higher than in the control group. This result parallels the fluorescence intensity of UCP2 immunoreactivity measured at day 6 (Fig 6C), and, as we suggest above, it can be explained by the fall-off of UCP2 induction with time. In contrast, this did not occur in the ghrelin-treated I/R group, probably because of the threefold injection of this peptide. The induction of UCP2 expression by ghrelin may be more rapid than that due to IPC, perhaps because it is receptor-mediated rather than by the release of ROS.

## Conclusion

These coherent data on ghrelin- and IPC-induced protection afford support for the hypothesis that UCP2 upregulation may often be implicated in different types of neuroprotection, and perhaps in the protection of other organs, although the present two strategies of neuroprotection clearly have a different time-course of action. The possible involvement of UCP2 in ghrelin-induced protection against ischemic lesion has not been previously explored. Our results add several in vivo lines of evidence connecting it to IPC protection. Although we did not establish functional data on UCP2 involvement in protection, our data encourage further study of the temporal aspects of its in vivo expression, together with an evaluation of its expression in other models of cerebral ischemia.

## Methods

### Animal preparation and general protocols

Male Wistar rats weighing 280-330 g were used. All procedures conformed to institutional guidelines for experiments on living animals. Four different types of living animal preparation were used as follows, based on the classical 4-vessel global forebrain ischemia model:

**1) ***I/R group*: Rats subjected to I/R were prepared by electrocoagulation of the vertebral arteries on day -1, then by clamping both common carotid arteries for 15 min under anesthesia on day 0. In each phase they were anesthetized with pentobarbital (50 mg/kg i.p.). Rectal temperature was maintained above 37.0°C. The efficiency of the ischemic manoeuver was appraised by recording the electrocorticogramme (Biopac Student Lab, Biopac Systems, Inc) and by observing the pupil dilation. The ischemia was accepted if the EEG became flat (isoelectric signal) within 1 min, and the pupil dilation attained 100%. Three days later (day 3), the animal was sacrificed for immunohistochemistry (IHC) or RT-PCR (see below).

**2) ***IPC+I/R group*: Rats were prepared in the same way for 4-vessel ischemia, but instead of 15 min, the first, preconditioning, ischemia lasted 3 min. On day 3, a second global ischemia was induced for 15 min, and three days later (day 6) the rats were sacrificed under halothane anesthesia.

**3) ***IPC group*: Rats were preconditioned only, by inducing, as described above, an ischemia lasting only 3 min on day 0, then sacrificing them on day 3.

**4) ***Control group *(CON): These rats were prepared by performing sham operations for vertebral coagulation and carotid clamping, then sacrificing them 3 days later.

The immunohistochemical comparison of the above 4 groups comprised experiment A1 (n = 4 animals per group), and the RT-PCR comparison comprised experiment A2 (n = 6 animals per group).

#### Experiments on the time-course of UCP2 expression (experiment B)

The results of experiment A led us to a supplementary experiment, designed to determine how much UCP2 expression persisted at 6 days after the preconditioning ischemia. Three new groups (n = 4 animals each) were prepared for comparison, i.e. a control group, an IPC group constituted as above, and an IPC group killed at day 6.

#### Experiments on ROS involvement (experiment C)

Since it has been shown that ROS scavenging reduces the protection induced by IPC [40], we carried out additional experiments to test the hypothesis that this effect may be due to the effect of ROS on UCP2 expression. We performed immunohistochemical evaluation of UCP2 expression on animals (n = 4 per group) subjected to preconditioning ischemia (same as 3-day IPC group), to which we administered superoxide dismutase (SOD, Sigma, 10000 U/kg i.p.) (SPC group) or vehicle (saline) (VPC group) 5 min before the brief ischemia. We killed the rats 3 days later for IHC.

#### Experiments using ghrelin treatment (experiment D)

In a recent study [34], we evaluated the effects of ghrelin administration on the number of lesioned pyramidal cells in the CA1 area from I/R rats in paraformaldehyde-fixed tissue by hemotoxylin and eosin staining. In the present work, we made preparations (n = 6 animals per group) for RT-PCR measurements of the amount of UCP2 mRNA in the same three groups as previously used, i.e. 1) *I/R group*, treated with vehicle; 2) *I/R +ghrelin group *(I/R+G); 3) *control group *treated with vehicle (CON). Ghrelin was obtained from AnaSpec, Inc. It was dissolved in distilled water (1 mg/ml) and stored at -20°C until use. For administration, it was diluted ×10 in 0.9% saline. Treated rats received 0.4 mg/kg i.p. three times, immediately after the ischemia, and 24 and 48 h later.

### Immunohistochemistry

#### General tissue treatment

All groups of rats were deeply anesthetized with halothane and transcardially perfused with heparinized ice-cold saline followed by 400 ml solution of 4% paraformaldehyde in phosphate-buffered saline (PBS), pH 7.4. The brains were post-fixed overnight in the same fixative, cryoprotected in 20% sucrose in PBS for 48 h, then frozen instantaneously in isopentane at -45°C. Coronal frozen sections (20 μm thick) cut on a Leica CM3050S cryotome were collected in PBS, mounted on Superfrost "Plus" slides (Menzel GmbH & Co KG, Braunschweig), and processed as follows. After treatment with 5% bovine serum albumin (BSA) plus 1% triton ×100 in PBS for 30 min, the sections were incubated for 48 h at 4°C in the primary antibody diluted in the same BSA/triton ×100 PBS solution. After several washes, secondary antibody incubation was performed in the same way for 24 h. In some experiments the sections were incubated with DAPI (10 μg/ml) for 15 min then rewashed. The sections were coverslipped using mowiol solution (prepared with N-propyl-gallate, pH 8). The primary antibodies used were: 1) goat anti-UCP2 C-terminus (sc-6525), or goat anti-UCP2 N-terminus (sc-6526) both from Santa Cruz Biotechnology, diluted ×200; 2) mouse anti-NeuN (MAB377, Chemicon), diluted ×250. The secondary antibodies were: 1) rabbit anti-goat Cy3 (C2821, Sigma) or rabbit anti-goat Alexa 555 (A-21431, Molecular Probes), diluted ×200; 2) goat anti-mouse Alexa fluor-488 (A-11029, Molecular Probes) diluted ×100. Negative controls, i.e. omission of primary antibodies, revealed no staining of either UCP2 or NeuN protein.

Positive controls of UCP2 staining were obtained by removing stomachs from untreated rats and rats which had fasted for 24 h. These tissues were fixed by transcardial perfusion of paraformaldehyde as described above, followed by the same IHC procedures.

#### Confocal microscopy

In each of the 3 immunohistochemical experiments (A1, B, C) in which intergroup comparison was intended, 4 runs (n = 4 animals per group) were made, each using one animal from each group. We thus obtained 4 totally independent comparisons. Equivalent sections from each animal of the different groups were processed under rigorously identical conditions. Several sets of comparisons were made as follows, using either a Bio-Rad MRC 600 (Bio-Rad Microscience, Hertfordshire, UK) with ×25 oil-immersion objective, or a Zeiss LSM 510 META confocal laser microscope (Zeiss, Oberkochen, Germany) with a Plan Neofluar 40× N.A.1.2 oil-immersion objective or a Plan Apochromat 63X N.A.1.4 oil-immersion objective using the LSM510 software v4.0 (Zeiss).

**1) **For UCP2 staining between the 4 basic groups (control, I/R, IPC+I/R, IPC), two sections from one animal of each of the four groups (a total of 8 sections) were used. These sections were taken at the level 5.7-5.2 mm anterior of the interaural line. The fluorophore-labelled sections were examined in a thick central optical plane. This manoeuver was performed bilaterally in two sites incorporating the pyramidal layer of each CA1 area (hence 4 sites/section). The confocal laser-scanning microscope settings used for quantification were identical for all images of any one series.

**2) **Similar procedures were used for the comparison of IPC groups subject to SOD or vehicle treatment (3 groups), and for the comparison of staining at day 3, day 6 and control. Sections of stomach were similarly compared after identical treatments of the fasted and control animals.

**3) **For counting of intact NeuN-stained CA1 neurons, 4 images were acquired from a hippocampal section from each brain cut at the same level as those used for UCP2 staining measurements.

#### Measurements of pyramidal cell staining intensity and lesions

After outlining the densely packed pyramidal layer, the intensity of CA1 UCP2 staining was calculated in arbitrary units by subtracting the background value (determined outside the pyramidal layer in the same image) from that of the defined pyramidal layer. For each of the 4 animals, the mean value obtained from the 8 images was used in the statistical comparison between groups. NeuN staining was used to count in a blinded manner the number of intact neurons/mm in the CA1 layer.

### UCP2 RT-PCR of rat hippocampus

On the appropriate day (see above), the rats were anesthetized with pentobarbital and sacrificed by decapitation. The brains were quickly removed, and the hippocampi dissected out, rapidly frozen in liquid nitrogen and stored at -80°C before use. The specimens were homogenized and total RNA was isolated using GIT (Guanidine Isothiocyanate) reagent. The first strand cDNA was synthesized from 1 μg of total RNA using oligo (dT) primer. PCR was carried out using 5 μl of cDNA template and the specific sense and antisense primers of β-actin and UCP2. The primer sequences used were:

β-actin forward, 5'-AAGATCCTGACCGAGCGTGG-3';

β-actin reverse, 5'-CAGCACTGTGTTGGCATAGAGG-3';

UCP2 forward, 5'-CTACAAGACCATTGCACGA-3';

UCP2 reverse 5'-CTCATAGGTGACAAACATTA-3'.

PCR thermal cycling parameters were as follows: 35 cycles of 94°C for 1 min, 58°C for 1 min, 72°C for 1 min. The PCR products obtained were loaded on to 2% agarose gels and evaluated by electrophoresis. The intensities of the amplified bands were estimated by 1D Image Analysis Software (Kodak, USA). The levels of UCP2 mRNA expression was normalised to that of β-actin mRNA.

### Statistical methods

The data are expressed as mean ± standard error (S.E.M). n indicates the number of animals in the groups. Comparisons of 3 or 4 groups were made by one-way analysis of variance (ANOVA) or a repeated measures ANOVA, followed by Tukey's test to determine intergroup differences. Distribution normality was checked. All analyses were performed using Sigma Stat or Statview software, and differences were considered to be statistically significant when p < 0.05.

## Abbreviations

I/R: ischemia/reperfusion; ROS: reactive oxygen species; IPC: ischemic preconditioning; UCP2: uncoupling protein-2; GHS: growth hormone secretagogue; GHS-R: growth hormone secretagogue receptor; CON: control; IHC: immunohistochemistry; RT-PCR: reverse transcriptase polymerase chain reaction; BSA: bovine serum albumin; PBS: phosphate buffered saline; DAPI: 4',6'-diamidino-2-phenyl indole; SOD: superoxide dismutase; MnSOD: manganese superoxide dismutase; Cu-ZnSOD: copper-zinc superoxide dismutase; GIT: guanidine isothiocyanate; ANOVA: analysis of variance; GH: growth hormone; mRNA: messenger ribonucleic acid; cDNA: complementary deoxyribonucleic acid; S.E.M.: standard error of the mean.

## Competing interests

The authors declare that they have no competing interests.

## Authors' contributions

YL was the principal investigator at the bench. She was associated with the experimental design, acquisition of data, analysis and interpretation, and preparation of the MS. LC conceived the original subject and strategy, made important contributions to the content of the study, and participated in the MS drafting. XX conceived and performed the RT-PCR experiments. EV supervised the overall study, providing important intellectual support and helped draft the MS. RS was the principal practical supervisor and conceptor and was associated with the experimental design, data acquisition and interpretation and the final version of the MS. All authors read and approved the final manuscript.
